# Enhanced intracellular delivery and antifungal potency of amphotericin B via PEG15HS-lipid nanoparticles

**DOI:** 10.1016/j.ijpx.2026.100605

**Published:** 2026-07-11

**Authors:** Kévin Brunet, Adrien Baillod, Jonathan Clarhaut, Henri Grau, Sandrine Marchand, Frédéric Tewes

**Affiliations:** a INSERM U1070 PHAR2, Poitiers, France; b Université de Poitiers, Faculté de médecine et pharmacie, Poitiers, France; c CHU de Poitiers, service de Mycologie-Parasitologie, Département des agents infectieux, Poitiers, France; d CHU de Poitiers, service de Toxicologie et de Pharmacocinétique, Poitiers, France

**Keywords:** Amphotericin B, Lipid nanoparticles, PEG15HS, *Cryptococcus neoformans*, Intracellular delivery, Antifungal formulation

## Abstract

**Background:**

*Cryptococcus neoformans* is responsible for life-threatening infections with persistently high mortality despite current amphotericin B (AmB)–based treatments. Although liposomal AmB (AmBisome®) remains the standard of care, its complex composition and high production cost limit accessibility and the development of generic versions. Improving AmB efficacy therefore represents a priority. We previously demonstrated that PEG-15 hydroxystearate (PEG15HS) enhances the antifungal activity of AmB by modulating its aggregation state toward more active, less toxic species. Building on these findings, we aimed to develop a simple and scalable lipid-nanoparticle (LNP) formulation combining AmB and PEG15HS to potentiate AmB activity against *C. neoformans.*

**Methods:**

AmB-loaded LNPs were produced by the phase-inversion temperature method using a Labrafac™ WL1349/tributyrin (1:1, *w*/w) oil phase selected for optimal AmB solubility and colloidal stability. Two particle sizes (∼40 nm and ∼ 100 nm) were obtained by varying the surfactant-to-oil ratio. Formulations were characterized physicochemically and evaluated *in vitro* against clinical *C. neoformans* isolates by MIC determination and time–kill curve analysis. Cellular localization was investigated by confocal and transmission electron microscopy.

**Results:**

Both AmB-LNP formulations markedly enhanced antifungal efficacy, reducing MICs up to 32-fold compared with liposomal AmB and demonstrating faster and more extensive fungicidal activity. Microscopy revealed that AmB-LNPs were internalized by *C. neoformans*, whereas blank LNPs remained extracellular, indicating that AmB may facilitate nanoparticle passage through the fungal cell wall and plasma membrane.

**Conclusion:**

PEG15HS-stabilized lipid nanoparticles substantially improve the antifungal performance of AmB, likely through controlled aggregation and enhanced interaction with fungal cells. This simplified, non-liposomal system offers a promising and more accessible alternative to AmBisome® for treating *C. neoformans* infections.

## Graphical Abstract

**Figure:**
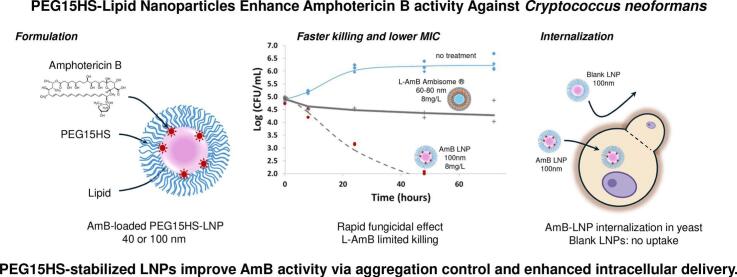

## 1 Introduction

Cryptococcosis is a severe invasive fungal infection caused by *Cryptococcus neoformans* complex and *C. gattii*, responsible for an estimated 200,000 deaths each year (Iyer et al., 2021a). This pathogen is considered a major global health threat and is listed in the WHO critical priority group of fungal pathogens (WHO, 2022). Cryptococcal infections mainly affect the brain in the form of meningitis, especially in immunocompromised patients, such as those with HIV or undergoing immunosuppressive therapies (Rajasingham et al., 2017). Nowadays, the first-line treatment in developed countries involves the combination of amphotericin B (AmB), formulated as liposomes (L-AmB), fluconazole and 5-fluorocytosine. This infection is difficult to treat, since the mortality rate under treatment remains 30%, regardless of the resource level of the healthcare system (May et al., 2016). In addition, liposomal AmB formulations such as AmBisome® are complex and costly to manufacture, which may limit accessibility and scalability. Therefore, there is a strong rationale for developing alternative formulations that are both simpler and capable of enhancing antifungal efficacy.

The mode of action of AmB is not fully elucidated, but its interaction with ergosterol is central to its antifungal activity (Carolus et al., 2020). Accumulating evidence indicates that AmB's therapeutic index, defined by its efficacy/toxicity ratio, depends on its aggregation state, which modulates its affinity for ergosterol versus cholesterol (Barwicz et al., 1992). Monomeric AmB is the most active and least toxic, whereas aggregated dimers increase toxicity due to enhanced cholesterol binding. In contrast, highly aggregated “superaggregates” are inactive but present minimal toxicity and may act as a reservoir for monomeric species (Zia et al., 2016).

We recently identified PEG15HS, a non-ionic surfactant used in FDA-approved parenteral and ophthalmic formulations (POLYOXYL, n.d.), as a promising modulator of AmB aggregation (Brunet et al., 2022). In vitro, PEG15HS markedly enhanced AmB activity against Mucorales, reducing minimum inhibitory concentrations (MICs) by up to 64-fold without increasing toxicity. This effect was associated with a shift in AmB's aggregation profile toward monomeric and highly aggregated non-toxic species (Brunet et al., 2022)*.*

Building on these findings, we explored the development of AmB-loaded lipid nanoparticles stabilized with PEG15HS for the treatment of cryptococcosis. Such a formulation could confer several advantages: synchronized pharmacokinetics of AmB and PEG15HS, improved control of the aggregation state of AmB, and ultimately an enhanced therapeutic index. This study investigates the potential of this novel nanoparticle-based strategy to improve AmB efficacy against *Cryptococcus* spp.

## 2 Materials and Methods

### 2.1 Lipid nanoparticle formulation and characterization

#### 2.1.1 Determination of amphotericin B solubility in oils.

To facilitate the encapsulation of AmB into lipid nanoparticles (LNPs), its solubility in different oils or oily mixtures was first evaluated. Stock solutions of AmB (5 mg/L) were prepared in methanol (Sigma-Aldrich, Missouri, USA). A volume corresponding to 2 mg of AmB was transferred into an Eppendorf® tube and the solvent was evaporated under a nitrogen stream using a nitrogen flow evaporator (Evaporator, Liebisch Labortechnik). An oil phase selected from various oils provided by Gattefossé SAS (France) (Table 1) was then added to the dried AmB residue and stirred at 850 rpm at 37 °C for 48 h using a Thermomixer HC (Starlab, Paris, France), a duration considered sufficient to reach solubility equilibrium. After incubation, samples were centrifuged at 14,000*g* for 10 min to remove undissolved material, and the supernatant was collected for AmB quantification (*n* = 2).

**Table 1:** List of oils used in this study.

Oil	Composition
Labrafac PG	Propylene glycol esters of caprylic and capric acids
Labrafac lipophile WL1349	Medium-chain triglycerides of caprylic and capric acids
Labrafil M 1944 CS	Mono-, di- and triglycerides and PEG-6 mono- and diesters of oleic acid
Peceol	Mono-, di- and triglycerides of oleic acid, with a predominant monoester fraction
Plurol oleic CC 497	Polyglyceryl-3 dioleate esters of oleic acid
Capryol 90 (propylene glycol monocaprylate)	Propylene glycol esters of caprylic acid composed mainly of monoesters
Tributyrin	Glyceryl tributyrate
Labrafac PG / Tributyrin (1:1, w/w)	Binary mixture
Labrafac Lipophile WL1349 / Tributyrin (1:1, w/w)	Binary mixture

For quantification, oil samples were diluted in dimethyl sulfoxide (DMSO), and absorbance was measured at 416 nm using a microplate reader (Infinite M200 PRO, TECAN). Calibration curves were prepared in DMSO over the concentration range of 5–50 mg/L (5, 10, 20, 30, 35, 40 and 50 mg/L). The analytical method was validated by assessing linearity (R^2^ > 0.99) and reproducibility across three independent calibration curves obtained on different days, with slope and intercept variations below 20%.

#### 2.1.2 Preparation of lipid nanoparticles.

LNPs with diameters below 150 nm were prepared using the phase inversion temperature (PIT) method as previously described (Salager et al., 2001; Anton et al., 2007; Valcourt et al., 2021). Briefly, the selected oil phase was mixed with a nonionic surfactant (PEG-15 hydroxystearate, Solutol® HS15, BASF, France) and 800 mg of Milli-Q water. The mixture was heated under mechanical stirring (80 °C, 1400 rpm) for 10 min and subsequently cooled to 25 °C under the same agitation conditions. This heating–cooling cycle was repeated once to refine nanoparticle size distribution. LNPs stability was evaluated by measuring particle size immediately after formulation and after 3 months of storage at 4 °C in the dark.

#### 2.1.3 Preparation of amphotericin B-loaded lipid nanoparticles

AmB-loaded LNPs were prepared using an oil phase composed of 50 wt% medium-chain triglycerides (Labrafac™) and 50 wt% tributyrin, containing 50 μg of AmB. This oil phase was mixed with the non-ionic surfactant PEGHS15 and 800 mg of Milli-Q water. The mixture was heated under mechanical stirring (80 °C, 1400 rpm) for 10 min and then cooled to 25 °C under the same agitation. The heating–cooling cycle was repeated once to refine the LNP size distribution. The Labrafac™/tributyrin mixture was selected as the oily phase as it provided an optimal compromise between enhanced AmB solubility (conferred by tributyrin) and nanoparticle stability (conferred by Labrafac™). LNPs were prepared using two different surfactant-to-oil ratios (SOR; 1.5 and 0.67) to obtain nanoparticles with different sizes. The formulation compositions and corresponding codes are presented in Table 2.

**Table 2:** Composition of nanoparticles formulated with or without amphotericin B.

Formulation	Oily phase (mg)	Solutol® (mg)	SOR	AmB (mg/L)
AmB-LNPs-40	80	120	1.5	50
Blank LNPs-40	80	120	1.5	0
AmB-LNPs-100	120	80	0.67	50
Blank LNPs-100	120	80	0.67	0

*Nanoparticles characterization.*

The size distribution of LNPs was determined by dynamic light scattering (DLS) using a Zetasizer NanoZS (Malvern Instruments, Orsay, France). LNP suspensions were diluted 1:20 (*v*/v) with Milli-Q water (resistivity at 25 °C: 18.2 MΩ·cm; viscosity: 0.894 mPa·s; refractive index: 1.335). Measurements were performed at 25 °C in triplicate. The *Z*-average diameter and polydispersity index (PDI) were recorded.

Nanoparticle morphology and size distribution were also examined by transmission electron microscopy (TEM) using a JEM-1010 microscope (JEOL, France) operating at 80 kV.

The zeta potential of LNPs was determined by electrophoretic light scattering (ELS) using a Zetasizer NanoZS (Malvern Instruments) at pH 3.4, 6.3 and 9.5. Measurements were performed at constant ionic strength (0.005 M), adjusted with NaCl solution (0.15 M). Mean zeta potential values were obtained from three measurements at 25 °C and plotted as a function of pH.

#### 2.1.4 Determination of amphotericin B association with lipid nanoparticles

The association of AmB with LNPs was evaluated by ultrafiltration using Amicon® Ultra centrifugal filters (100 kDa molecular weight cut-off, Millipore, France). AmB-LNP suspensions containing 8 to 10 μg of AmB were loaded onto the filtration device and centrifuged at 5000 ×*g* for 15 min. The filtrate was collected and the retained fraction was washed three times with PBS using the same centrifugation conditions. The initial filtrate and washing fractions were pooled and evaporated at 35 °C under a gentle nitrogen stream. Samples were subsequently reconstituted in DMSO and analyzed by UV–visible spectrophotometry at 416 nm using a calibration curve prepared in DMSO.

To evaluate non-specific adsorption of AmB to the filtration membrane, a free AmB solution containing the same amount of drug was processed under identical ultrafiltration conditions. Additional recovery controls were performed to assess potential losses during sample handling, evaporation, and reconstitution. The fraction of AmB associated with LNPs (*F*) was calculated from the amount of drug recovered in the filtrate relative to the initial amount loaded onto the filter, taking into account the recovery obtained in control experiments.

*F* = $1-\frac{\text{Amount of}\;\mathrm{AmB}\;\text{filtrate from}\;\mathrm{LNP}}{\text{Amount Recovered from free}\;\mathrm{AmB}\;\text{solution control}}$

#### 2.1.5 Fungal strains and media

Seven clinical strains of *Cryptococcus neoformans* (C1–C7, six serotypes A and one serotype AD) isolated from blood, cerebrospinal fluid or bronchoalveolar lavage samples were used. Isolates were stored at −80 °C and cultured on Sabouraud agar (BioMérieux, France) at 37 °C before use. RPMI-1640 medium (Sigma-Aldrich, Saint-Quentin-Fallavier, France) supplemented with 2% (*w*/*v*) dextrose and buffered with 0.165 mol/L MOPS to pH 7 was used for minimum inhibitory concentration (MIC) determination and time-kill curve experiments. The medium was sterilized by filtration through a 0.22 μm membrane.

#### 2.1.6 Minimum inhibitory concentration

Minimum inhibitory concentrations (MICs) of AmB, liposomal AmB ((L-AmB); Ambisome®, Gilead, Foster City, CA, USA), blank-LNPs, and AmB-loaded LNPs were determined according to the European Committee on Antimicrobial Susceptibility Testing (EUCAST) guideline E.DEF 7.3.2 (Arendrup et al., 2020). AmB concentration ranged from 0.008 to 4 mg/L. Yeasts were grown for 24–48 h on YPD agar at 30 °C and suspended in sterile water to obtain a final inoculum of 10^5^ CFU/mL. In 96-well microplates, 100 μL of fungal suspension was added to 100 μL of twofold-concentrated RPMI medium containing twofold-concentrated AmB. Plates were incubated at 30 °C for 48 h, and absorbance at 530 nm was measured using a microplate reader (Infinite M200 PRO, TECAN, Lyon, France). MIC values were defined as the lowest drug concentration inhibiting ≥90% of fungal growth compared with the untreated control and were measured in duplicate.

#### 2.1.7 Time-kill curve experiments

Time-kill curve (TKC) experiments were performed with AmB-loaded LNPs and L-AmB. *C. neoformans* cells were grown overnight in liquid RPMI at 30 °C. A 2 mL suspension containing 10^5^ CFU/mL and AmB concentrations ranging from 0.125 to 8 mg/L was added to the wells of 24-well plates and incubated for 72 h at 30 °C. At 0, 4, 8, 24, 48, and 72 h, 100 μL samples were collected, serially diluted tenfold, and plated on YPD agar. After 48 h of incubation, colonies were counted and fungal concentrations were expressed as CFU/mL. All experiments were performed in duplicate.

#### 2.1.8 Confocal microscopy

The interaction of LNPs or Ambisome® liposomes (L-AmB) with yeast cells were investigated by confocal laser scanning microscopy (CLSM). *C. neoformans* cells were cultured overnight in liquid RPMI at 30 °C. LNPs and L-Amb were labeled in red with the lipophilic fluorescent dye DiL (Thermo Fisher Scientific, France), and yeast cells were stained in green with CMFDA (CellTracker™ Green, Thermo Fisher Scientific, France). Green-labeled yeast cells were incubated for 18 h at either 4 °C or 37 °C with red-labeled AmB-loaded LNPs, red-labeled blank LNPs, or unlabeled blank LNPs. In parallel, green-labeled yeast cells were incubated with red-labeled L-AmB for 18 h at 37 °C. After incubation, cells were washed three times with 0.1 M PBS (pH 7.4) to remove non-associated nanoparticles and transferred to 8-well μ-slide chambers (Ibidi, Munich, Germany). Images were acquired using a FluoView FV-3000 confocal microscope (Olympus, Rungis, France) equipped with a 100× oil immersion objective. *Z*-stack images (1024 × 1024 pixels; field size 127 × 127 μm) were collected with a z-step of 0.5 μm for each condition and reconstructed as 3D projections using IMARIS software.

#### 2.1.9 Transmission electron microscopy

Internalization of LNPs was further examined by TEM. Yeast cells were cultured overnight in RPMI at 30 °C and treated for 18 h with AmB-loaded or blank LNPs. After washing, cells were fixed overnight at 4 °C in 2.5% glutaraldehyde in 1 M PBS (pH 7.1). Samples were washed three times in PBS and post-fixed with 1% osmium tetroxide for 1 h. Following fixation, samples were dehydrated through graded ethanol solutions (70–100%) and embedded in Spurr resin via progressive acetone/resin infiltration. Ultrathin sections (70–90 nm) were obtained using an Ultramicrotome UC6 (Leica) and examined with a transmission electron microscope operating at 80 kV.

## 3 Results

### 3.1 Nanoparticle development and amphotericin B solubility

To guide the selection of the oily phase for AmB-loaded lipid nanoparticles, the apparent solubility of AmB was quantified in a panel of pharmaceutically acceptable oils and selected binary mixtures (Table 3). Marked differences were observed across excipients, consistent with known polarity variations among lipid classes (Mu et al., 2013). Tributyrin, which belongs to the more polar short-chain triglycerides, provided the highest AmB solubility (336.55 ± 32.59 mg/L). Other relatively polar lipid excipients such as Plurol oleic CC 497 (a polyglyceryl ester) and Peceol (glyceryl mono-oleate) also solubilized AmB efficiently (172.69 ± 3.83 mg/L and 153.35 ± 20.68 mg/L, respectively). In contrast, low-polarity medium-chain excipients such as Labrafac lipophile WL1349 and Labrafac PG displayed very limited solubilization capacity (6.92 ± 3.2 mg/L and 9.34 ± 7.8 mg/L). Labrafil M1944 CS, which has intermediate polarity, showed moderate solubility (27.28 ± 12.77 mg/L), while Capryol 90 remained poorly solubilizing (15.19 ± 1.4 mg/L). Binary mixtures of tributyrin with Labrafac lipophile WL1349 or Labrafac PG (1:1, *w*/w) resulted in markedly reduced AmB solubility (34.22 ± 16.5 mg/L and 30.62 ± 10.39 mg/L), compared to pure tributyrin, consistent with a decrease in overall polarity when blending excipients of different chemical classes, as previously described by Mu et al. (Mu et al., 2013).

**Table 3:** Apparent solubility of amphotericin B (AmB) in pure oils and selected binary oily mixtures at room temperature. Values represent mean concentrations (mg/L) ± standard deviation of two independent measurements. Binary mixtures were prepared at a 1:1 mass ratio (*w*/w).

Oil	Concentration (mg/L)
Labrafac PG	9.34 ± 7.8
Labrafac lipophile WL1349	6.92 ± 3.2
Labrafil M1944 CS	27.28 ± 12.77
Plurol oleic CC 497	172.69 ± 3.83
Peceol	153.35 ± 20.68
Capryol 90	15.19 ± 1.4
Tributyrin	336.55 ± 32.59
Labrafac PG/Tributyrin (1/1; m/m)	30.62 ± 10.39
Labrafac lipophile WL 1349/Tributyrin (1/1; m/m)	34.22 ± 16.5

#### 3.1.1 Control of nanoparticle size through surfactant/oil ratio (SOR) modulation and evaluation of storage stability

To evaluate the capacity of the different oil phases to form nanoparticles with controlled dimensions, the surfactant/oil ratio (SOR) was systematically varied. Two SOR conditions were selected to generate distinct size populations: a high SOR (120 mg Solutol® / 80 mg oil) to promote the formation of smaller nanoparticles (∼50 nm), and a lower SOR (80–90 mg Solutol® / 110–120 mg oil) to obtain larger particles (> 100 nm). After establishing the feasibility of producing these two populations, their physical stability was assessed after 3 months of storage at 5 °C (Table 4).

**Table 4:** Hydrodynamic diameter (median, nm) and polydispersity index (PDI) of lipid nanoparticles formulated with different oil phases and oil/Solutol® ratios, measured immediately after preparation and after 3 months of storage at 5 °C. Two target size ranges (∼40–50 nm and ∼ 100–140 nm) were pursued by modulating the oil/surfactant ratio. Values represent mean ± standard deviation of four independent measurements.

Oil phase (mg)	Solutol® (mg)	Size (nm)	Size (nm) 3 months	PDI	PDI 3 months
Labrafac lipophile WL1349 (80 mg)	120	44.6 ± 1.6	44.5 ± 0.2	0.13 ± 0.03	0.18 ± 0.01
Labrafac lipophile WL1349 (110 mg)	90	141.5 ± 3.8	139.4 ± 0.2	0.15 ± 0.02	0.12 ± 0.01
Labrafac PG (80 mg)	120	36.2 ± 3.0	118.6 ± 1.6	0.05 ± 0.01	0.13 ± 0.01
Labrafac PG (120 mg)	80	113.0 ± 3.7	301.0 ± 1.1	0.15 ± 0.02	0.50 ± 0.16
Tributyrin (80 mg)	120	118.7 ± 11.4	184.3 ± 44.4	0.16 ± 0.01	0.24 ± 0.02
Tributyrin (120 mg)	80	222.6 ± 10.0	347.2 ± 5.3	0.11 ± 0.10	0.41 ± 0.16
Labrafac lipophile WL1349 /Tributyrin (80 mg, 1/1; m/m)	120	39.7 ± 0.4	44.5 ± 0.3	0.06 ± 0.02	0.08 ± 0.01
Labrafac lipophile WL1349 /Tributyrin (120 mg, 1/1; m/m)	80	103.4 ± 13.4	103.9 ± 0.5	0.13 ± 0.01	0.14 ± 0.01

Size modulation through SOR adjustment was effective but strongly dependent on the nature of the oil phase. For Labrafac lipophile WL1349 and the Labrafac WL1349/tributyrin (1:1, *w*/w) blend, increasing the SOR consistently produced nanoparticles in the 40–45 nm range, whereas decreasing the SOR yielded particles between 100 and 140 nm. These results show that both systems allow robust and predictable tuning of particle size across a well-defined window.

In contrast, tributyrin alone did not enable access to ∼50 nm particles, even at high SOR. The smallest tributyrin-based formulation reached 118.7 ± 11.4 nm, and decreasing the SOR further increased the diameter to 222.6 ± 10.0 nm. Thus, although particle size remained sensitive to SOR changes, the accessible size range was intrinsically shifted toward larger dimensions when tributyrin was used as the sole oily phase.

After 3 months of storage at 5 °C, clear differences in colloidal stability were observed across formulations. Nanoparticles prepared with Labrafac PG or with tributyrin showed substantial increases in mean particle size and marked elevation of PDI values, particularly under low-SOR conditions, indicating a loss of size uniformity over time. In contrast, Labrafac lipophile WL1349-based formulations remained stable at both SOR levels, with minimal variation in particle diameter and consistently low PDIs. The Labrafac WL1349/tributyrin 1:1 (*w*/w) mixture also maintained its initial size distributions over the storage period while preserving effective SOR-dependent size modulation. Overall, these results show that while SOR determines the initial nanoparticle size, long-term size maintenance is highly dependent on the physicochemical properties of the oil phase.

#### 3.1.2 Formulation and physicochemical characterization of AmB-loaded lipid nanoparticles

Based on the initial screening, the binary oil mixture Labrafac™ lipophile WL1349/tributyrin (1:1, *w*/w) was selected as the optimal oily phase, offering a balance between enhanced AmB solubility (tributyrin) and long-term colloidal stability (Labrafac™ WL1349). AmB-loaded lipid nanoparticles (AmB-LNPs) were prepared using this mixture at two surfactant/oil ratios (SOR = 1.5 and 0.67) to generate two distinct size populations. Amphotericin B was incorporated at a concentration corresponding to saturation of the lipid phase, with 50 μg of AmB dispersed in 200 mg of total lipid (Labrafac™ WL1349/tributyrin 1:1, *w*/w) in the presence of Solutol®. Formulation compositions and physicochemical characteristics are reported in Table 5.

**Table 5:** Composition and physicochemical characteristics of lipid nanoparticles formulated with the Labrafac™ WL1349/Tributyrin (1:1, *w*/w) oil phase at two surfactant/oil ratios (SOR), with or without amphotericin B (AmB). Hydrodynamic diameter (median, nm) and polydispersity index (PDI) of AmB-loaded formulations are expressed as mean ± standard deviation (*n* = 4).

AmB-nanoparticles	Oil phase	Solutol® (mg)	SOR	AmB (μg)	Size (nm)	PDI
AmB-LNPs-40	Labrafac lipophile WL1349/Tributyrin (80 mg, 1/1; m/m)	120	1.5	50	37,9 ± 0,9	0,06 ± 0,01
AmB-LNPs-100	Labrafac lipophile WL1349/Tributyrin (120 mg, 1/1; m/m)	80	0.67	50	103,4 ± 13,4	0,40 ± 0,05

Although AmB has extremely low aqueous solubility, its amphiphilic nature raises the possibility of partial distribution between the oily core and the aqueous phase. However, number-weighted size distributions showed no additional peaks that would correspond to free AmB aggregates or micelles, despite the very low aqueous solubility and sub-milligram aggregation onset (≈ 0.55 mg/L) of AmB (Tancréde et al., 1990). The absence of sub-10 nm species therefore indicates that AmB remained associated with the nanoparticles rather than forming independent aggregates. This interpretation was further supported by ultrafiltration experiments using a 100 kDa membrane. Following separation of LNPs-associated and non-associated drug fractions, only 28% of the initial AmB was recovered in the filtrate, corresponding to an apparent LNPs-associated fraction of approximately 72%. Control experiments using free AmB were performed to account for non-specific membrane adsorption and procedural losses.

At an SOR of 1.5, AmB-LNPs-40 exhibited a mean hydrodynamic diameter of 37.9 ± 0.9 nm with a low polydispersity index (PDI 0.06 ± 0.01), consistent with a narrow and homogeneous population. At an SOR of 0.67, AmB-LNPs-100 displayed a larger mean diameter (103.4 ± 13.4 nm) and a broader size distribution (PDI 0.40 ± 0.05), reflecting reduced colloidal uniformity at lower surfactant content.

**Fig. 1:**
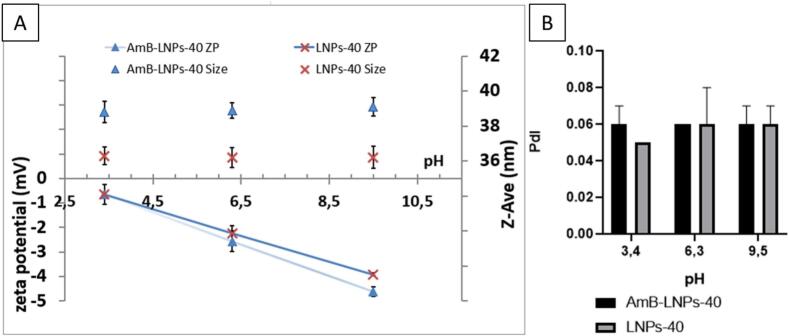
pH-dependent physicochemical characterization of ∼ 40 nm lipid nanoparticles formulated with the Labrafac™ WL1349/tributyrin oil phase. (A) Hydrodynamic diameter (*Z*-Ave) and zeta potential of AmB-LNPs-40 (blue triangles) and blank LNPs-40 (red crosses) measured by DLS and ELS, respectively, across pH 3.4–9.5. (B) Polydispersity index (PDI) determined by DLS at the corresponding pH values. Data are presented as mean ± SD (*n* = 3).

To examine whether AmB could partially localize at the nanoparticle interface, the influence of pH on the physicochemical properties of AmB-LNPs-40 was assessed. Because surface charge reflects the ionization state of interfacial groups, monitoring the zeta potential as a function of pH can provide indirect insight into AmB exposure at or near the particle surface. The hydrodynamic diameter, polydispersity index (PDI), and zeta potential of AmB-LNPs-40 were measured over a pH range of 3.4 to 9.5 and compared with blank LNPs-40 (Fig. 1). Particle size remained stable across the entire pH range for both formulations, indicating that pH variations did not induce aggregation, swelling, or other structural changes. PDIs also remained low, confirming preservation of a narrow and homogeneous size distribution. In contrast, zeta potential exhibited a clear pH-dependent trend (Fig. 1A). Both blank and AmB-loaded nanoparticles became progressively more negative with increasing pH. At pH 3.4, the two formulations showed comparable near-neutral zeta potentials (approximately −0.65 ± 0.39 mV). At higher pH values, however, AmB-LNPs-40 displayed slightly more negative surface charges than blank LNPs-40. At pH 9.5, the zeta potential reached −4.62 ± 0.19 mV for AmB-LNPs-40 compared with −3.92 ± 0.06 mV for blank particles. The modest but reproducible difference between the two formulations indicates that AmB contributes to nanoparticle surface charge behavior. Although this observation is consistent with partial exposure of AmB at or near the nanoparticle interface, the magnitude of the effect does not allow definitive conclusions regarding the precise localization of the drug within the nanoparticles.

**Fig. 2:**
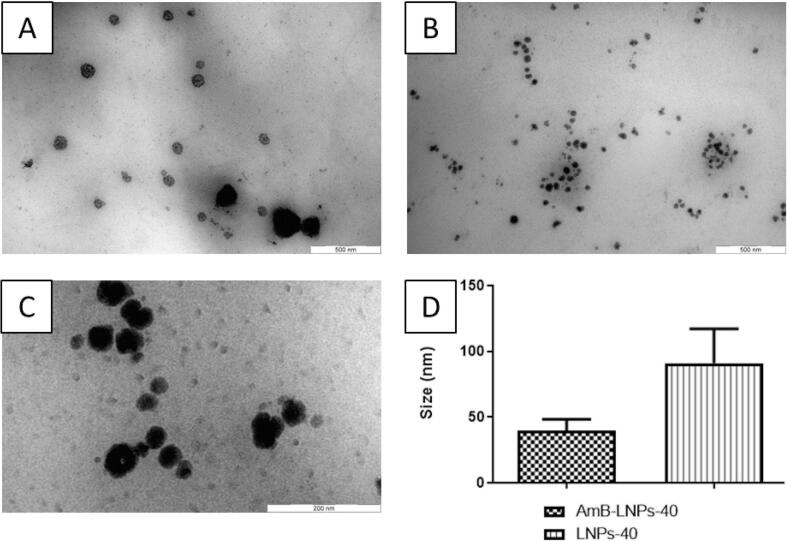
Transmission electron microscopy (TEM) characterization of blank LNPs-40 and AmB-LNPs-40. (A) LNPs-40 observed at 20,000× magnification. (B) AmB-LNPs-40 observed at 20,000× magnification. (C) AmB-LNPs-40 observed at 80,000× magnification. (D) Mean particle size determined from TEM image analysis (40–70 particles per formulation). Data are presented as mean ± SD.

The morphology of the nanoparticles, which exhibited a mean Z-average diameter of approximately 40 nm by DLS, was examined by transmission electron microscopy (Fig. 2). Micrographs acquired at 20,000× magnification (Fig. 2A–B) showed predominantly spherical, electron-dense particles with well-defined contours for both blank LNPs-40 and AmB-LNPs-40. Clear differences were observed between the two formulations. AmB-LNPs-40 appeared smaller and more homogeneous, with less size variability than blank LNPs-40. This difference was even more apparent at higher magnification (80,000×; Fig. 2C), where AmB-LNPs-40 displayed uniformly distributed particles with narrow size dispersion, whereas blank LNPs-40 showed larger and more polydisperse structures.

Quantitative analysis of 40–70 particles per formulation confirmed these observations (Fig. 2D). AmB-LNPs-40 had a mean TEM diameter of 39.6 ± 8.6 nm, consistent with particles in the same size range as those measured by DLS. In contrast, blank LNPs-40 displayed a significantly larger mean diameter of 91.1 ± 26.0 nm, approximately 2.3-fold greater than AmB-LNPs-40 and with a noticeably broader size distribution. Overall, TEM analysis confirmed the spherical morphology of the nanoparticles and demonstrated that AmB-loaded formulations produced smaller and more uniform particles than blank LNPs-40.

### 3.2 In vitro antifungal activity against *Cryptococcus neoformans*

The antifungal activity of AmB-loaded lipid nanoparticles was evaluated against seven clinical strains of *Cryptococcus neoformans* and compared with free AmB and liposomal AmB (L-AmB). MIC values are presented in Table 6. Blank LNPs prepared without AmB did not exhibit detectable antifungal activity at any tested concentration, including carrier concentrations equivalent to those present in formulations containing up to 256 mg/L AmB. MIC determinations were highly reproducible between replicates, with variability not exceeding one two-fold dilution step. Free AmB consistently exhibited an MIC of 0.5 mg/L across all strains, whereas L-AmB showed higher MICs ranging from 1 to 2 mg/L. In contrast, both AmB-LNP formulations, prepared with mean particle diameters of approximately 38 nm and 103 nm, demonstrated markedly enhanced antifungal activity, with MICs ranging from 0.06 to 0.25 mg/L depending on the strain. When expressed as the ratio of L-AmB MIC to AmB-LNP MIC, this corresponded to an 8- to 32-fold increase in potency for AmB-LNPs-40 and a 4- to 16-fold increase for AmB-LNPs-100. Importantly, the enhanced activity was consistently observed across all tested strains and for both nanoparticle formulations. Overall, the two nanoparticle size populations exhibited comparable antifungal efficacy.

**Table 6:** Minimum inhibitory concentrations (MICs) of amphotericin B (AmB), liposomal amphotericin B (L-AmB), and AmB-loaded lipid nanoparticles (∼40 nm and ∼ 100 nm) against seven clinical strains of *Cryptococcus neoformans* (*n* = 2). The L-AmB MIC/LNP MIC ratio reflects the fold improvement in activity of nanoparticle formulations relative to L-AmB.

Strains	MIC (mg/L)				L-AmB MIC over LNPs MIC ratio	
	AmB	L-AmB	AmB-LNPs-40	AmB-LNPs-100	AmB-LNPs-40	AmB-LNPs-100
C1	0.5	2	0.06	0.125	32	16
C2	0.5	1	0.06	0.06	16	16
C3	0.5	2	0.125	0.125	16	16
C4	0.5	1	0.125	0.25	8	4
C5	0.5	2	0.25	0.25	8	8
C6	0.5	2	0.25	0.25	8	8
C7	0.5	2	0.25	0.25	8	8

**Fig. 3:**
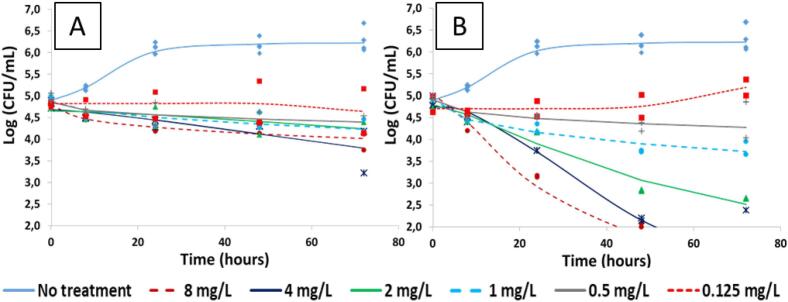
Time–kill curves of *Cryptococcus neoformans* strain C5 in the presence of liposomal amphotericin B (A) or AmB-LNPs-40 (B) at concentrations of 0.125, 0.5, 1, 2, 4, and 8 mg/L. The tested concentration range encompasses the respective MIC values (2 mg/L for L-AmB and 0.25 mg/L for AmB-LNPs-40). Points represent individual CFU counts, and solid lines represent fitted mean trends.

The fungicidal activity of AmB-LNPs-40 was further assessed by time–kill curve (TKC) analysis against strain C5 and compared with L-AmB over a concentration range of 0.125 to 8 mg/L (Fig. 3). This range encompassed the MIC values of both formulations (0.25 mg/L for AmB-LNPs-40 and 2 mg/L for L-AmB), enabling evaluation of killing kinetics at sub-MIC, MIC, and supra-MIC levels. Comparable killing profiles were observed between independent replicates. Across the tested concentration range, however, clear differences in pharmacodynamic activity were observed between the two formulations. L-AmB produced only a modest, concentration-dependent reduction in fungal burden, whereas AmB-LNPs-40 displayed a steeper and more pronounced concentration–response curve. CFU counts decreased more rapidly and to a greater extent with the nanoparticle formulation across the tested concentrations.

At concentrations between 2 and 8 mg/L, AmB-LNPs-40 induced a rapid and substantial decline in viable yeast cells, approaching near-complete eradication at the latest sampling times. In contrast, L-AmB showed slower and less extensive fungicidal activity, even at concentrations equal or exceeding its MIC. For example, at 2 mg/L, the fungal burden was reduced by 66.2% after 72 h with L-AmB, whereas the same concentration of AmB-LNPs-40 resulted in a 99.3% reduction over the same period. These results demonstrate that AmB-LNP40 not only reduced MIC values but also enhanced the rate and extent of fungal killing compared with L-AmB.

### 3.3 Interaction of lipid nanoparticles with *Cryptococcus neoformans* cells

**Fig. 4:**
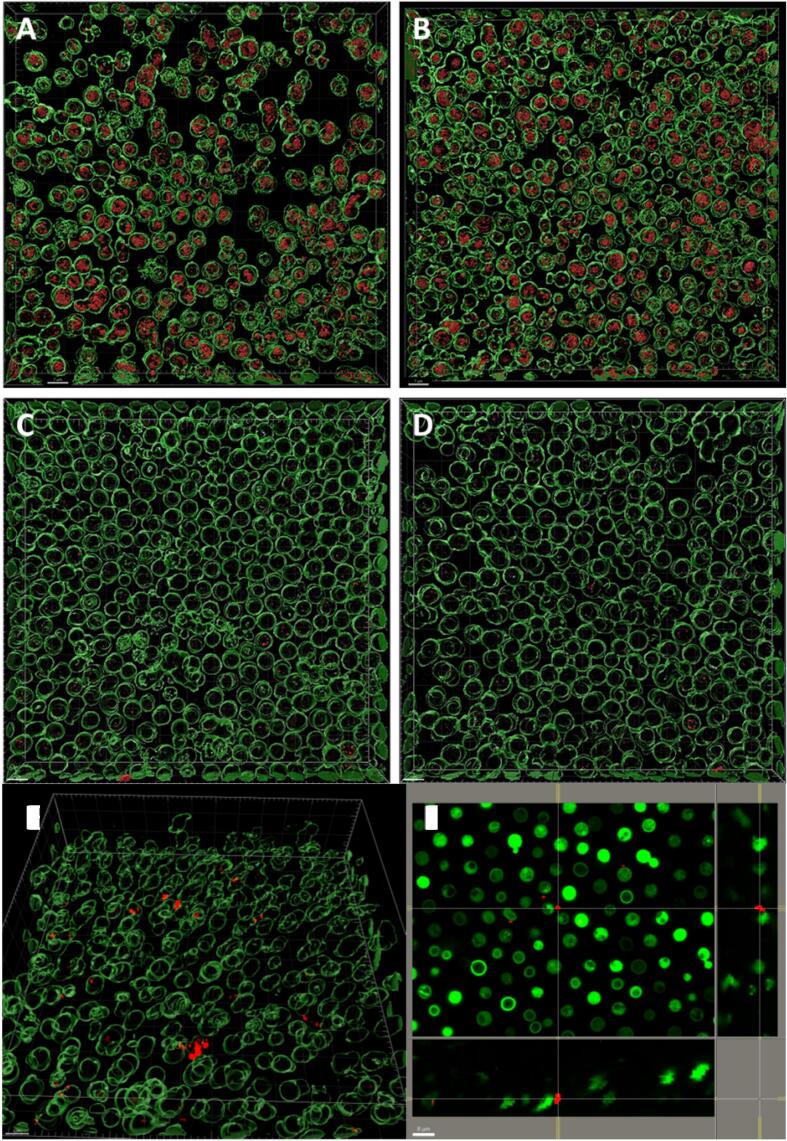
Confocal laser scanning microscopy (CLSM) analysis of the interaction between fluorescently labeled amphotericin B-loaded lipid nanoparticles (AmB-LNPs) and *Cryptococcus neoformans* after 18 h of incubation. Yeast cells were stained with CMFDA (green), while lipid nanoparticles and AmBisome® liposomes were labeled with DiI (red). Representative 3D projections show (A) AmB-LNP40 and (B) AmB-LNP100. Red fluorescence was detected both at the cell surface and within the cellular boundaries, indicating nanoparticle association and intracellular uptake by *C. neoformans*. In contrast, (C) blank LNP40 (without AmB) and (D) untreated cells exhibited only minimal background red fluorescence, confirming the specificity of the intracellular DiI signal observed in panels A and B. (E) 3D reconstruction of *C. neoformans* cells incubated with DiI-labeled AmBisome® liposomes. (F) Corresponding orthogonal x–z and y–z sections show that the red fluorescence is mainly located outside the yeast cells and concentrated at discrete sites on the cell surface, indicating surface association and local aggregation of liposomes rather than intracellular accumulation. Scale bars = 8 μm. (For interpretation of the references to colour in this figure legend, the reader is referred to the web version of this article.)

The interaction between AmB-LNPs and *Cryptococcus neoformans* was investigated using confocal laser scanning microscopy (CLSM) and transmission electron microscopy (TEM) to determine nanoparticle localization within the cells. Following 18 h exposure to fluorescently labeled nanoparticles, the spatial distribution of LNPs relative to yeast cells was examined by CLSM. Nanoparticles were labeled with the lipophilic red dye DiI, while yeast cells were stained with CMFDA (green). *Z*-stack images were acquired and reconstructed as three-dimensional projections (Fig. 4). After incubation with both AmB-LNPs-40 (Fig. 4A) and AmB-LNPs-100 (Fig. 4B), red fluorescence was detected not only at the cell periphery but also within the boundaries of the green-stained yeast cells. Blank LNPs (Fig. 4C) or LNPs without DiL labeling (Fig. 4D) produced only minimal background signal, whereas AmB-LNPs-40 and AmB-LNPs-100 generated a markedly stronger and more homogeneous intracellular red signal. The persistence of intracellular fluorescence after extensive washing indicates that a fraction of the nanoparticle population was internalized during the 18 h incubation period. Similar localization patterns were observed for both nanoparticle sizes.

As a comparator, *C. neoformans* cells were incubated with DiI-labeled L-AmB. In contrast to AmB-LNPs, L-AmB-associated fluorescence remained predominantly confined to the cell surface and frequently appeared as discrete aggregates attached to the yeast cell wall (Fig. 4E–F). Orthogonal projections revealed little evidence of intracellular liposomal accumulation, suggesting that AmBisome® liposomes remained primarily associated with the outer cell surface under the conditions tested.

To investigate whether nanoparticle uptake required active cellular processes, CLSM experiments were additionally performed after incubation at 4 °C. AmB-LNP-associated intracellular fluorescence was still observed at low temperature, with a localization pattern comparable to that obtained at 37 °C (Supplementary material). These observations suggest that nanoparticle internalization is not exclusively dependent on energy-driven uptake mechanisms and may involve direct interactions between the nanoparticles and the fungal cell envelope.

**Fig. 5:**
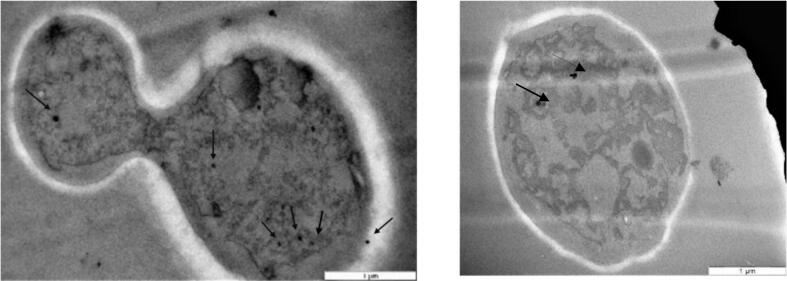
Transmission electron micrographs of non-contrasted *Cryptococcus neoformans* after 18 h treatment with AmB-LNPs-40 labeled with iodinated oil (AmB concentration: 0.5 mg/L). Ultrathin sections (70–90 nm) were analyzed; within these slices, 2–6 electron-dense structures per section were typically observed, indicating a relatively high local nanoparticle concentration. These electron-dense structures, corresponding to labeled nanoparticles, are visible within the cytoplasmic region, consistent with intracellular localization without evident organelle-specific accumulation.

To further investigate nanoparticle localization at higher resolution, TEM analysis was performed after 18 h treatment with AmB-LNPs-40 labeled with iodinated oil to enhance contrast (Fig. 5). Electron-dense structures consistent with labeled nanoparticles were observed within the yeast cytoplasm. These structures were detected inside the cellular boundaries rather than exclusively at the cell surface, supporting intracellular localization. However, nanoparticles did not appear to preferentially accumulate within specific organelles, and no clear compartmental enrichment was identified. Together, CLSM and TEM observations indicate that, after 18 h incubation, LNPs were associated with *C. neoformans* cells and that a fraction of the nanoparticles was localized intracellularly for both tested size populations.

## 4 Discussion

*Cryptococcus neoformans* is a major opportunistic fungal pathogen responsible for severe invasive infections, causing an estimated 200,000 deaths annually. The persistently high mortality rate, despite antifungal therapy, reflects limited drug efficacy associated with available treatments. The pathogen is therefore included in the WHO list of fungal priority pathogens, highlighting the urgent need for improved antifungal strategies. In this context, we investigated PEG15HS-based lipid nanoparticles (LNPs) as a formulation strategy to enhance the antifungal activity of amphotericin B (AmB).

### 4.1 Formulation strategy and physicochemical aspects

Oil screening confirmed that AmB solubility is highly dependent on oil polarity: tributyrin provided the highest solubilization capacity, whereas Labrafac™ lipophile WL1349 ensured superior colloidal stability. Their combination (1:1, *w*/w) offered a balanced compromise between solubility and long-term stability. By adjusting the surfactant/oil ratio (SOR), two well-defined nanoparticle populations (∼40 nm and ∼ 100 nm) were reproducibly obtained. Incorporation of AmB at saturation (50 μg per 200 mg lipid phase) did not impair nanoparticle formation or generate detectable free micellar aggregates, despite the very low aqueous solubility and sub-milligram aggregation threshold (≈ 0.55 mg/L) of AmB (Tancréde et al., 1990). This observation suggests that AmB remained associated with the nanoparticle system under the selected formulation conditions. Ultrafiltration studies further supported this interpretation, indicating that a large fraction of AmB remained associated with the LNPs fraction after correction for non-specific recovery losses.

Although the AmB concentration achieved in the present formulation (∼0.34 mg/mL) is lower than that obtained after reconstitution of liposomal AmB formulations such as AmBisome® (∼4 mg/mL), the objective of this work was not to maximize drug loading. Rather, the formulation was designed to modulate the physicochemical state of AmB and its interaction with fungal cells. This distinction is important because antifungal activity depends not only on the amount of drug incorporated into the formulation but also on its aggregation state, accessibility to fungal membranes, and cellular distribution. The markedly improved MIC values and fungicidal activity observed with AmB-LNPs compared with L-AmB indicate that antifungal performance can be enhanced without necessarily increasing drug loading. Furthermore, the formulation concentration (340 mg/L) remains well above clinically achieved plasma concentrations of amphotericin B and exceeds the MICs observed for all tested isolates by several orders of magnitude.

Dynamic light scattering (DLS) and transmission electron microscopy (TEM) provided complementary information on nanoparticle size. DLS measures hydrodynamic diameters in suspension, whereas TEM characterizes particles after deposition and drying on a grid. Blank LNPs appeared larger by TEM than by DLS, likely reflecting deformation or spreading of the soft lipid matrix during drying. In contrast, AmB-LNPs-40 showed good agreement between the two techniques, suggesting that incorporation of AmB may produce a more compact nanoparticle structure less prone to deformation.

The pH-dependent study was performed to assess whether incorporation of AmB modified nanoparticle surface properties. Although the absolute differences in zeta potential between blank LNPs and AmB-LNPs remained limited, AmB-LNPs consistently exhibited more negative surface charges at higher pH values, corresponding to an approximately 18% increase in charge magnitude at pH 9.5. These findings suggest that AmB contributes to the interfacial charge behavior of the nanoparticles. However, zeta potential measurements alone cannot establish the precise localization of AmB and should therefore be regarded as supportive rather than definitive evidence of drug exposure at the nanoparticle interface. While the present work focused on aqueous formulations, future pharmaceutical development will require assessment of long-term storage stability. Although freeze-drying and spray-drying were not investigated here, related lipid nanocapsule and nanoemulsion-based systems have been successfully converted into dry powders with preservation of their physicochemical properties following reconstitution (Friis et al., 2023; Li et al., 2011). This suggests that solid-state formulation of AmB-LNPs may be feasible, although dedicated studies will be required to confirm this.

### 4.2 Enhanced antifungal activity and potential mechanisms

Both AmB-LNP formulations markedly improved antifungal activity against *C. neoformans*, reducing MIC values by up to 32-fold compared with liposomal AmB (L-AmB). However, MIC values for AmB against *C. neoformans* do not always correlate with fungicidal activity (Rodero et al., 2000). Time-kill experiments were therefore performed to better evaluate pharmacodynamic effects.

L-AmB produced only gradual reductions in fungal burden even at concentrations equal to or above its MIC. In contrast, AmB-LNPs-40 exhibited faster and more pronounced killing kinetics. At 2 mg/L, L-AmB reduced the inoculum by approximately 66% after 72 h, whereas AmB-LNPs-40 achieved approximately 99% reduction under the same conditions. These results indicate that PEG15HS-based LNPs enhance not only apparent potency but also fungicidal dynamics. Because AmB-LNPs and L-AmB differ in their drug-to-lipid ratios, equivalent AmB concentrations correspond to different lipid concentrations and nanoparticle numbers. However, blank LNPs did not exhibit detectable antifungal activity in the present study, and PEGHS15 alone was previously reported to be inactive against filamentous fungi at substantially higher concentrations (Brunet et al., 2022). These observations suggest that the enhanced antifungal activity of AmB-LNPs results from formulation-dependent modulation of amphotericin B activity rather than from the presence of larger amounts of carrier material.

Two complementary mechanisms may account for this enhancement. First, control of the AmB aggregation state by PEG15HS may play a central role. Previous work demonstrated that PEGHS15 modulates the aggregation equilibrium of AmB, stabilizing it primarily in monomeric and non-toxic super-aggregated forms that exhibit higher affinity for ergosterol and reduced binding to cholesterol (Brunet et al., 2022). In the present system, co-localization of AmB and PEG15HS within the same nanoparticles may maintain the drug in these pharmacologically favourable aggregation states.

Second, enhanced interaction with fungal cells may contribute to the improved antifungal activity. Confocal microscopy and TEM showed that AmB-loaded LNPs (∼40 nm and ∼ 100 nm) were associated with *C. neoformans* cells and that a fraction of nanoparticles was detected within the cytoplasm after 18 h of incubation. Given that the estimated pore size of the yeast cell wall (∼6 nm) should restrict diffusion of particles of this size (Walker et al., 2018), these findings suggest that AmB may facilitate nanoparticle translocation across the cell wall and plasma membrane.

Walker et al. previously reported that AmBisome liposomes (60–80 nm) can traverse the cell walls of *Candida albicans* and *Cryptococcus neoformans* as intact vesicles and reach the plasma membrane (Walker et al., 2018). Consistent with these observations, AmB-loaded LNPs were also able to cross the yeast cell wall. However, our CLSM experiments showed that, in contrast to AmBisome® liposomes, which remained predominantly associated with the yeast surface and displayed limited evidence of intracellular accumulation, AmB-LNPs were detected within the cytoplasmic compartment. Blank LNPs lacking AmB were not observed inside yeast cells, indicating that the presence of AmB is required for nanoparticle internalization. Nevertheless, because AmBisome® also contains AmB but remained largely confined to the cell surface, the presence of the drug alone cannot fully explain the distinct intracellular localization of AmB-LNPs. Rather, these findings suggest that the presentation of AmB within the nanoparticle structure may play a critical role. The zeta potential data are consistent with partial localization of AmB at or near the nanoparticle interface, raising the possibility that surface-accessible AmB promotes interactions with the fungal cell envelope and facilitates nanoparticle translocation across cellular barriers. TEM images further indicated that internalized nanoparticles were dispersed throughout the cytoplasm without preferential localization to specific organelles. Together, these findings suggest that both the presence of AmB and its physicochemical organization within PEG15HS-based LNPs contribute to enhanced cellular penetration and intracellular delivery compared with conventional liposomal formulations.

PEG15HS is a non-ionic surfactant listed as an inactive ingredient in the FDA database and widely used as a solubilizer in marketed oral, parenteral, and ophthalmic formulations. Consistent with its established role as a pharmaceutical excipient, blank LNPs did not exhibit detectable antifungal activity in the present study, and PEGHS15 alone was previously shown to be inactive against *Mucorales* at concentrations up to 1024 mg/L (Brunet et al., 2022). Its favourable safety profile and biocompatibility make it an attractive excipient for antifungal drug delivery (Ali, 2019; US FDA, 2022). Moreover, resistance of *C. neoformans* to antifungal agents has been reported (Iyer et al., 2021b). The use of a non-antimicrobial excipient such as PEG15HS as a potentiator is unlikely to directly promote resistance selection, and combining an antimicrobial agent with a non-antimicrobial potentiator may reduce the probability of resistance emergence compared with combinations involving multiple active drugs (Schneider et al., 2017).

Several limitations of this study should be acknowledged. First, although previous spectroscopic studies have demonstrated that PEG15HS influences the aggregation state of amphotericin B, the aggregation state of the drug was not directly characterized in the final nanoparticle formulation investigated here. Second, while CLSM and TEM demonstrated intracellular localization of AmB-loaded nanoparticles, the precise mechanism responsible for nanoparticle translocation across the fungal cell envelope remains incompletely understood. Although intracellular LNPs-associated signal was observed, these experiments do not distinguish intact LNPS from AmB released after cellular entry. Finally, all efficacy studies were performed in vitro, and future pharmacokinetic, safety, and in vivo efficacy studies will be required to determine the translational potential of this formulation. Despite these limitations, the consistent improvements in antifungal potency, fungicidal activity, and intracellular delivery support the potential of PEG15HS-based lipid nanoparticles as a promising alternative amphotericin B delivery platform.

### 4.3 Toward inhaled and topical amphotericin B for non-cerebral cryptococcosis

Beyond cryptococcal meningitis, cryptococcosis frequently presents as pulmonary and, less commonly, cutaneous disease. Management of these non-cerebral forms remains largely extrapolated from systemic therapies, with limited formulations specifically optimized for local delivery. While Amphotericin B deoxycholate (e.g., Fungizone®) is available as a topical suspension for cutaneous use, and L-AmB (AmBisome®) can be administered off-label via nebulization, these approaches are not specifically designed for efficient or stable local delivery. In particular, liposomal systems may be susceptible to shear forces and air–liquid interface stresses during aerosolization, potentially leading to vesicle disruption or drug leakage unless engineered for aerosol robustness. In contrast, LNPs, characterized by a dense lipid matrix and non-vesicular internal structure, may offer enhanced mechanical stability under such conditions. These physicochemical features support the rationale for developing LNP-based amphotericin B formulations tailored for inhaled or topical administration. Such approaches could improve local drug retention, reduce systemic exposure, and ultimately expand therapeutic options for pulmonary and cutaneous cryptococcosis.

Although the present study demonstrates enhanced antifungal activity of AmB-LNPs, the optimal dosing strategy remains to be established. Because formulation-dependent changes in cellular interaction and antifungal potency may alter the exposure–response relationship compared with existing AmB formulations, dedicated pharmacokinetic and pharmacodynamic studies will be required to define appropriate dosing regimens. Such investigations will be particularly important for local delivery approaches, including pulmonary and topical administration, where drug distribution and retention are expected to differ markedly from systemic administration.

## CRediT authorship contribution statement

**Kévin Brunet:** Writing – review & editing, Validation, Methodology, Conceptualization. **Adrien Baillod:** Writing – original draft, Investigation, Formal analysis. **Jonathan Clarhaut:** Investigation, Formal analysis. **Henri Grau:** Methodology. **Sandrine Marchand:** Writing – review & editing, Project administration. **Frédéric Tewes:** Writing – review & editing, Supervision, Methodology, Conceptualization.

## Declaration of competing interest

The authors declare the following financial interests/personal relationships which may be considered as potential competing interests:

Frédéric Tewes and Kévin Brunet have patent “A composition comprising lipid nanoparticles and an antifungal polyene” issued to INSERM. If there are other authors, they declare that they have no known competing financial interests or personal relationships that could have appeared to influence the work reported in this paper.

## Data Availability

Data will be made available on request.

## Appendix A Supplementary data

Supplementary Figure S1. Uptake of DiI-labeled lipid nanoparticles (LNPs) by *Cryptococcus* *neoformans* cells visualized by confocal laser scanning microscopy (CLSM). Cryptococcus cells were labeled with CellTracker™ CMFDA (green), and LNPs were labeled with DiI (red). Cells were incubated with LNPs for 18 h at either 37°C or 4°C before CLSM imaging. Representative images (top) and corresponding higher-magnification views (bottom) show a similar distribution of DiI fluorescence under both incubation conditions, suggesting that LNP uptake is not substantially affected by incubation at 4°C.
