# Supplementary figures and images for: Enhanced intracellular delivery and antifungal potency of amphotericin B via PEG15HS-lipid nanoparticles
Source: Int J Pharm X. 2026 Jul 11;12:100605. doi: 10.1016/j.ijpx.2026.100605 (PMC13401022; doi:10.1016/j.ijpx.2026.100605)

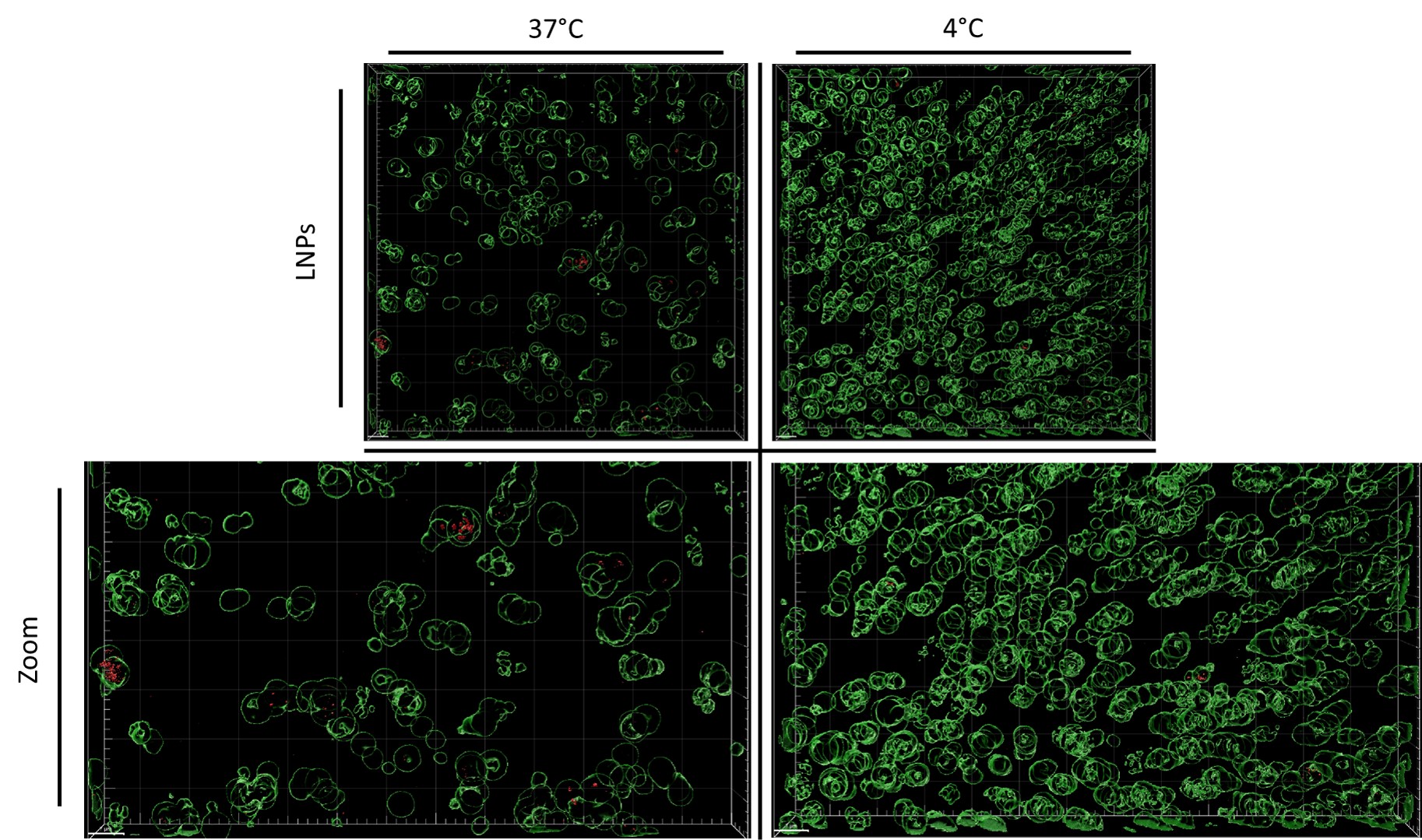

Supplement: Supplementary Figure S1. Uptake of DiI-labeled lipid nanoparticles (LNPs) by Cryptococcus neoformans cells visualized by confocal laser scanning microscopy (CLSM). Cryptococcus cells were labeled with CellTracker™ CMFDA (green), and LNPs were labeled with DiI (red). Cells were incubated with LNPs fo [file mmc1.zip › SupplementaryMaterial_V2.jpg]
